# Effects of Irradiation Dose and O_**2**_ and CO_**2**_ Concentrations in Packages on Foodborne Pathogenic Bacteria and Quality of Ready-to-Cook Seasoned Ground Beef Product (Meatball) during Refrigerated Storage

**DOI:** 10.1100/2012/274219

**Published:** 2012-04-01

**Authors:** Gurbuz Gunes, Neriman Yilmaz, Aylin Ozturk

**Affiliations:** Food Engineering Department, Istanbul Technical University, Maslak, 34469 Istanbul, Turkey

## Abstract

Combined effects of gamma irradiation and concentrations of O_2_ (0, 5, 21%) and CO_2_ (0, 50%) on survival of *Escherichia coli* O157:H7, *Salmonella enteritidis*, *Listeria monocytogenes*, lipid oxidation, and color changes in ready-to-cook seasoned ground beef (meatball) during refrigerated storage were investigated. Ground beef seasoned with mixed spices was packaged in varying O_2_ and CO_2_ levels and irradiated at 2 and 4 kGy. Irradiation (4 kGy) caused about 6 Log inactivation of the inoculated pathogens. Inactivation of *Salmonella* was 0.9- and 0.4-Log lower in 0 and 5% O_2_, respectively, compared to 21% O_2_. Irradiation at 2 and 4 kGy increased thiobarbituric acid reactive substances in meatballs by 0.12 and 0.28 mg malondialdehyde kg^−1^, respectively, compared to control. In reduced-O_2_ packages, radiation-induced oxidation was lower, and the initial color of an irradiated sample was maintained. Packaging with 0% + 50% CO_2_ or 5% O_2_ + 50% CO_2_ maintained the oxidative and the color quality of irradiated meatballs during 14-day refrigerated storage. MAP with 5%O_2_ + 50% CO_2_ combined with irradiation up to 4 kGy is suggested for refrigerated meatballs to reduce the foodborne pathogen risk and to maintain the quality.

## 1. Introduction

Consumer demand for minimally processed ready-to-cook and ready-to-eat food products has been increasing throughout the world. Ready-to-cook meat products such as ground beef seasoned with various spices and herbs in the form of meatballs or patties are very popular among consumers. However, potentially dangerous pathogens including Shiga toxin producing *Escherichia coli* O157:H7, *Salmonella,* and *Listeria monocytogenes* in these products can create a great risk of foodborne diseases [1]. In fact, there have been a number of foodborne-disease outbreaks associated with consumption of meat products contaminated with these pathogens in the last couple of decades [2–4].

Gamma irradiation is an effective cold decontamination process approved for beef in many countries including USA and Turkey. Effective inactivation of pathogens and shelf life extension of meat products by irradiation have been reported in a number of studies [5–9]. Irradiation below 3 kGy doses significantly decreased populations of *E. coli *O157:H7,* Salmonella*, and *L. monocytogenes* in meat and poultry [6, 10]. Irradiation increased 2-thiobarbituric acid-reactive substances (TBARSs) in different meat products under different conditions [11, 12]. Color is an important quality attribute that influences consumer acceptance of meat or meat products. Red color of beef products can be lost, turning into brown or gray upon irradiation [13]. Therefore, the irradiation dose applied to inactivate the target microorganisms is often limited by the induced degradations in the organoleptic qualities of the products.

Modified atmosphere packaging (MAP) involves enclosure of a product in a package atmosphere different from air [14]. Elevated CO_2_ and reduced O_2_ levels are commonly used to extend shelf-life of food products through inhibition of microbial growth and oxidative changes [14, 15]. Use of O_2_-free atmospheres in packages has been suggested for different meat products [15]. However, there is a risk of *C. botulinum* growth in anaerobic packages [10]. Thus, inclusion of a low level of O_2_ in packages can be suggested. MAP containing 5% O_2_ with elevated CO_2_ did not adversely affect the quality of meatball compared to anaerobic MAP and was suggested for meatball [16].

The combination of preservation technologies can have synergistic effects on microbial inactivation and may allow the use of milder treatments to control their undesirable effects on the quality of food products. Combination of MAP and gamma irradiation can be a useful approach to inactivate pathogenic microorganisms and maintain the quality of ready-to-cook meatball. Benefits of the combined effects of MAP and irradiation have been shown in different products including turkey meat, minced pork, and ground beef [8, 17, 18]. The atmospheric composition around the product during irradiation can affect radiation-induced changes in the product, and thus it needs to be investigated [19]. Therefore, the effects of reduced O_2_ levels on quality of irradiated meatball compared to O_2_-free (anaerobic) and ambient atmosphere (air) would be useful for determination of optimum packaging atmosphere for the product.

The objective of this study was to investigate the combined effects of O_2_ and CO_2_ concentrations in packages and gamma irradiation on the survival of *E. coli *O157:H7, *S. enteritidis*, and *L. monocytogenes*, and lipid oxidation and color changes in ready-to-cook seasoned ground beef (meatball) during refrigerated storage.

## 2. Material and Methods

### 2.1. Materials

Beef, salt, black pepper, cumin, red pepper, onion powder, garlic powder, and bread powder were purchased from a local supermarket. MacConkey agar (SMAC) with 5-bromo-4-chloro-3-indoyl-b-d-glucuronide (SMAC-BCIG), Cefixime Tellurite (CT), peptone, and Dryspot *E. coli* O157:H7 latex agglutination test were purchased from Oxoid (Hampshire, UK). Xylose lysine desoxycholate (XLD) agar, Oxford Listeria selective agar, Listeria selective supplement, tryptic soy agar (TSA), and thiobarbituric acid (TBA) were purchased from Merck (Darmstadt, Germany); Butylated hydroxytoluene (BHT), 1,1,3,3-Tetraethoxypropane (TEP), and trichloroacetic acid (TCA) were purchased from Fluka (Buschs, Switzerland). Low-density polyethylene (LDPE) film (oxygen permeability: 3800 cc/m^2^·day·atm at 0°C 0% RH) and a multilayered polyethylene teraphylate/polyethylene-ethylene vinyl alcohol-polyethylene (PET/PE-EVOH-PE) film (oxygen permeability 1.2 cc/m^2^.day.atm at 0°C 0% RH) were donated by Korozo Packaging Company GmbH (Istanbul, Turkey).

### 2.2. Preparation of Meatballs

The surface of beef was sterilized with ethanol and the residual ethanol was burned-off before the beef was ground through a plate with 4.7 mm pores using a meat grinder (Moulinex HV4, France) in our laboratory. Total fat content of the ground beef was measured as 14% on average. Meatballs were prepared with ground beef seasoned with the following dry ingredients: bread powder (8%), onion powder (3%), red pepper (2%), cumin (2%), salt (2%), garlic powder (0.5%), and black pepper (0.1%). All the ingredients were mixed and kneaded by hand with sanitized gloves for about 30 minutes and left in a refrigerator at 3 ± 1°C for one night. Then the seasoned ground beef was shaped into small pieces (18 ± 3 g) using a disc-shaped mould (3 cm diameter × 1 cm height) before packaging.

### 2.3. Preparation of Inoculums and Inoculation of Meatball Samples

*S. enteritidis *KUKEN 369, *L. monocytogenes *SLCC 2371, and* E. coli *O157:H7 (ATCC 700728) in TSA slants were suspended in peptone (0.1%) and spread on TSA plates and incubated at 35°C for 24 hours. A loop of the cultures was transferred to TSA again and incubated for another 24 hours. A loop of the cultures from the TSA plates was added to test tubes with 0.1% peptone water and vortexed to prepare separate stock cultures of each pathogen with approximately 10^8^ CFU mL^−1^ concentration using McFarland standard turbidity tubes. A 0.2 mL from each stock culture was added to the center of the pieces of meatballs to get a final population of 10^6^ CFU g^−1^ for each pathogen.

### 2.4. Packaging and Irradiation of Samples

Meatballs were packaged in a low barrier LDPE film for aerobic packaging (21% O_2_ + 0% CO_2_) and a high barrier PET/PE-EVOH-PE film for MAP. The packaging treatments were the following combinations of O_2_ and CO_2_: 0% O_2_ + 0% CO_2_, 0% O_2_ + 50% CO_2_, 5% O_2_ + 0% CO_2_, 5% O_2_ + 50% CO_2_, 21% O_2_ + 50% CO_2_, and 21% O_2_ + 0% CO_2_. The proportion of the gas components were completed to 100% with N_2_ when needed. The gas mixtures were made by mixing O_2_, CO_2_, and N_2_ in a gas mixer (PBI Dansensor Map Mix 9000, Denmark) connected to a packaging machine (Multivac C200, Germany). Packages were sealed after flushed with the gas mixtures using the packaging machine. Gas compositions in the gas mixtures and the packages were measured using a gas analyzer (PBI Dansensor CheckMate, Denmark).

The packaged samples were irradiated at a commercial food irradiation plant (Gamma-Pak GmbH., Cerkezkoy, Turkey) with a ^60^Co source at an average sample temperature of 9 ± 3°C in refrigerated boxes. The samples were exposed to gamma rays at a rate of 1.33 kGy h^−1^ for 1.5 and 3 hours amounting to a cumulative dose of 2 and 4 kGy, respectively. The dose rate was measured by Amber 3042 dosimeters (Harwell Dosimeters Ltd, Oxfordshire, UK). Unirradiated (0 kGy) control samples were kept outside the irradiation chamber during the treatments. The experiments were carried out in two replicates. All the samples were stored at 3 ± 1°C for 14 days after the irradiation treatments. Gas compositions in packages, *E. coli* O157:H7, *S. enteritidis, *and *L. monocytogenes *counts, TBARS, and color were determined on day 1, day 7, and day 14 during storage.

### 2.5. Enumeration of the Pathogens

*E. coli *O157:H7, *S. enteritidis*, and *L. monocytogenes *were enumerated using Thin Agar Layer method [20]. Selective media used for these 3 organisms were SMAC-BCIG with CT supplement, XLD agar, and Oxford Listeria selective agar with Listeria selective supplement, respectively. Petri plates with double agar layer were prepared 1 day before the day of analyses as follows: 14 mL of the selective media was poured into a petri plate. Then a 14 mL nonselective TSA was added onto the solidified selective media layer. Meatball samples (18 ± 3 g each) diluted in sterile peptone water (0.1%) were homogenized at medium speed for 2 minutes using a stomacher (Seward 400, Seward Ltd, London, UK). Homogenized samples were serially diluted and spread-plated onto the double layer agar plates. The plates were incubated at 35°C for 24–48 hours and typical colonies were counted. Representative *E. coli* O157:H7 colonies (one to two colonies from some plates in each experiment) were verified with the Dryspot latex agglutination test.

### 2.6. Measurement of Lipid Oxidation

Lipid oxidation was determined by the TBARS method [21]. A 5% trichloroacetic acid (TCA) solution in water and a 7.2% BHT solution in ethanol were prepared. A 10 g of sample was mixed with 34 mL of the TCA and 1 mL BHT solutions and homogenized at 11200 rpm for 2 min using a homogenizer (IKA-Ultra-Turrax T18, Germany). The homogenized sample was filtered through a filter paper (Whatman No. 4) into a 50 mL flask and diluted to the volume with the TCA solution. Next, 5 mL of the solution was transferred to a test tube, mixed with 5 mL of 0.02 M thiobarbituric acid (TBA) solution, and incubated at 80°C in a water bath for 20 min. Absorbance was measured at 532 nm against a blank containing 5 mL of TBA and 5 mL of 5% TCA using a spectrophotometer (T80 UV/VIS, PG Instrument, UK). Standard curve was prepared using 1,1,3,3-Tetraethoxypropane (TEP) as the malondialdehyde (MDA) standard at different concentrations. The TBARS values were expressed as mg MDA kg^−1^ of samples.

### 2.7. Color Measurements

Color (Hunter L, a, b values) of the samples was measured using a Chroma meter (CR-400, Konica Minolta, Japan). The instrument was calibrated with a standard white reflector plate prior to the measurements. The measurements were performed on each piece of meatball at five different locations and averaged.

### 2.8. Statistical Analyses

The data were subjected to analysis of variance and Tukey pairwise comparison using a statistical software (Minitab Inc., State College, PA, USA). Regression equation (Log CFU g^−1^ = A × Dose + B) was fitted to the microbiological data, and D_10_-value was calculated as the negative reciprocal of the slope (D_10_ = −1/A). All statistical analyses were evaluated at *α* = 0.05 significance level.

## 3. Results

Gas compositions of the packages were determined periodically during storage. There were small reductions in O_2_ and small increase in CO_2_ levels in all the packages possibly due to microbial activities and permeation through the packages (data not shown). However, these changes can be considered negligible because the gas concentrations remained close to the initial target levels during storage, and there were no overlaps among different gas levels targeted in packaging treatments.

### 3.1. Growth/Survival of the Pathogens

The initial population of the pathogens in meatballs were significantly reduced by gamma irradiation (*P* < 0.001). Irradiation at 4 kGy resulted in about 6 Log inactivation of the pathogens, and their counts dropped below the detection limit (<10^2^ CFU g^−1^) in all samples (Tables 1 and 2). *E. coli *O157:H7 was more sensitive to irradiation than *L. monocytogenes* and *S. enteritidis*. The *E. coli *O157:H7 count in the samples irradiated at 2 kGy was below the detection limit during 14-day storage. D_10_-values for the pathogens were calculated using day 1 data presented in Tables 1 and 2 and reported for each O_2_ level in the package headspace (Table 3). These values tend to decrease with increased O_2_ levels, but the differences were not statistically significant. The inactivation *S. enteritidis* by irradiation was 0.54 and 1.2 Log higher at 5% and 21% O_2_, respectively, compared to 0% O_2_ as assessed from day 1 data (Tables 1 and 2). On the other hand, no effects of O_2_ on the radiation-induced inactivation of *L. monocytogenes *were detected. The growth of the three pathogens in unirradiated samples during storage was slightly higher (0.07–0.25 Log difference) in 21% O_2_ than 0 and 5% O_2_ (*P* < 0.05), while no difference between the effect of 0% and 5% O_2_ was detected.

Carbon dioxide did not affect the radiation-induced inactivation of pathogens. No inhibitory effects of CO_2_ on number of *L. monocytogenes* and *S. enteritidis* in both the irradiated and the unirradiated samples were detected during 14-day storage. However, the 50% CO_2_ resulted in 0.12 Log lower *E. coli *O157:H7 counts, on average, in unirradiated samples during storage (*P* < 0.05).

### 3.2. Lipid Oxidation

**Figure 1:**
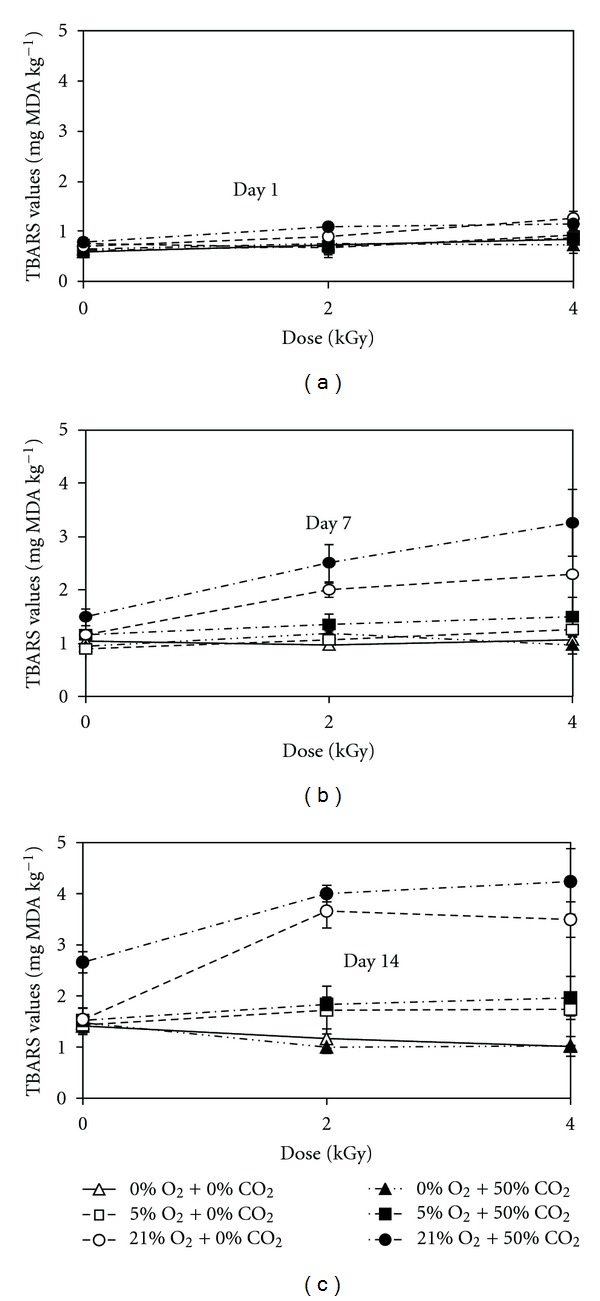
Effects of irradiation dose and O_2_ and CO_2_ levels in packages on TBARS values of ready-to-cook meatballs during storage at 3°C. Error bars represent standard deviations.

TBARS values of the meatballs increased significantly as the irradiation dose increased (Figure 1). Irradiation at 2 and 4 kGy resulted in, respectively, 0.12 and 0.28 mg MDA kg^−1^ higher TBARS values on day 1 compared to the unirradiated (0 kGy) samples (*P* < 0.01). These effects became more pronounced with 0.36 and 0.42 mg MDA kg^−1^ higher TBARS in the samples irradiated at 2 and 4 kGy, respectively, than in the unirradiated samples on day 14 of storage. There was a significant interaction of O_2_ and irradiation dose. Irradiation at 2 and 4 kGy resulted in 0.26 and 0.42 mg MDA kg^−1^ higher TBARS values, respectively, in the presence of 21% O_2_ compared to 0% O_2_ on day 1 (*P* < 0.01). The elevated CO_2_ (50%) in the package headspace did not affect the radiation-induced TBARS values.

As the O_2_ concentration in the packages increased, the TBARS values increased significantly (*P* < 0.01). Oxygen levels of 0% and 5% in the packages resulted in 1.15 and 0.9 mg MDA kg^−1^ lower TBARS values, respectively, compared to 21% O_2_ during 14-day storage (Figure 1). The difference between the TBARS values due to 0 and 5% O_2_ was relatively small (0.25 mg MDA kg^−1^) during 14-day storage. The elevated CO_2_ (50%) resulted in slightly higher (0.186 mg MDA kg^−1^) TBARS value during 14-day storage, but its effect was not significant at the low O_2_ levels (Figure 1).

### 3.3. Color

**Figure 2:**
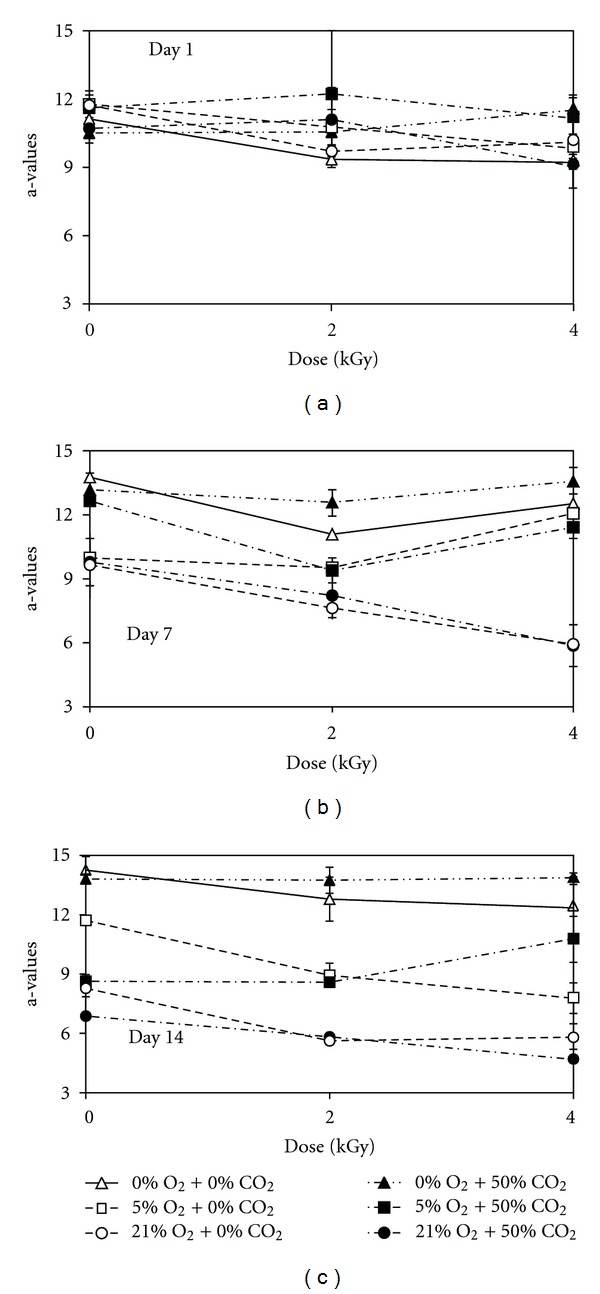
Effects of irradiation dose and O_2_ and CO_2_ levels in packages on a-values of ready-to-cook meatballs during storage at 3°C. Error bars represent standard deviations.

In general, there were no changes in a-values in the first 7-day storage, but the a-values decreased significantly after 14-day storage. Irradiation at 2 and 4 kGy resulted in significant reduction (*P* < 0.001) in the a-values of the meatballs after 7-day storage (Figure 2). Average a-value of the irradiated (2 and 4 kGy) meatball was 9.9, while it was 13.1 in the unirradiated (0 kGy) samples after 7-day storage. No difference between the effect of 2 and 4 kGy was detected. As the O_2_ concentration decreased, smaller reduction in the a-values was observed (Figure 2). Average a-value of the samples in the packages with 21% O_2_ was 7.8, while it was 10.8 in the 5% O_2_-containing packages after 7-day storage. The packages with no O_2_ maintained the initial a-values of the meatballs during storage (Figure 2). Carbon dioxide had no effect on the a*-*values of the samples. L- and b-values of the samples were in the range of 29.1–34.7 and 9.1–13.1, respectively, and they were not affected by the irradiation dose and O_2_ and CO_2_ levels.

## 4. Discussion

Irradiation effectively inactivated the pathogenic microorganisms in the meatball in all the packaging conditions tested. The inactivation was higher in the packages containing 21% O_2_. The D_10_-values for the pathogens in our study were in general agreement with the literature which reported D_10_-values for *S. enteritidis*, *L. monocytogenes*, and *E. coli *O157:H7 in various conditions as 0.51–0.71, 0.45–0.50, and 0.24–0.41 kGy, respectively [6, 10, 18, 19]. Differences in the reported D_10_-values in the literature for the same pathogens are probably due to the differences in the irradiation conditions (temperature, atmosphere, dose rate) and the product formulations, since these factors significantly affect the radiation-induced inactivation of microorganisms [5, 9, 10, 19].

Although 21% O_2_ in the packages resulted in higher radiation-induced inactivation of the pathogens, the difference between the number of pathogens in the 21% O_2_ and the 5% O_2_ packages was relatively small (approximately 0.2 Log). On the other hand, the lower O_2_ concentration in the packages maintained the oxidative and the color stability of the irradiated samples.

The oxidative and the color qualities of the irradiated meatballs were best maintained in the 0% O_2_ packages. However, the absence of O_2_ in the packages can create a great risk of anaerobic pathogens such as *C. botulinum* spores which have a high inherent radiation resistance (D_10_ = 2–3 kGy) [22]. Thus, the irradiation dose applied up to 4 kGy in this study would cause only 1-2 Log reduction in the spore count. Growth and toxin production by *C. botulinum *in packaged meat products have been detected at O_2_ levels below 2% [15]. The differences between the effects of 0 and 5% O_2_ on the quality attributes of the meatballs were negligible in our study. Therefore, we suggest that the meatball packages should contain 2–5% O_2_ during storage for the safety assurance of the product.

In conclusion, the safety risk due to the *L. monocytogenes*,* E. coli *O157:H7, and *S. enteritidis* in ready-to-cook meatballs can be significantly reduced by gamma irradiation. More than 6 Log reduction in *L. monocytogenes *and *S. enteritidis* by 4 kGy and 5 Log reduction in* E. coli *O157:H7 by 2 kGy were achieved in the meatballs. Lower irradiation doses would likely be sufficient for the products in commerce since the contamination levels would be lower than 5-6 Log. The low O_2_ (0% and 5%) levels inhibited the irradiation-induced color change and the oxidation significantly during refrigerated storage. An inclusion of 5% O_2_ in the packages is suggested as it did not adversely affect the quality of the irradiated meatballs compared to 0% O_2_. Although 50% CO_2_ did not affect the radiation-induced inactivation of the pathogens, it can inhibit growth of the surviving pathogens and other microorganisms in meatballs during refrigerated storage. Thus, MAP with 5% O_2_ + 50% CO_2_ combined with irradiation up to 4 kGy is suggested for refrigerated meatballs to reduce the foodborne pathogen risk and to maintain the quality. 

## Figures and Tables

**Table 1:** Changes in* S. enteritidis*, *L. monocytogenes*, and *E. coli *O157:H7 counts (Log CFU g^−1^) in ready-to-cook meatballs during storage at 3°C as affected by irradiation dose (kGy) and O_2_ levels in 0% CO_2_ containing packages.

Storage (day)	O_2_*	*S. enteritidis*			*L. monocytogenes*			*E. coli* O157:H7		
	(%)	0 kGy	2 kGy	4 kGy	0 kGy	2 kGy	4 kGy	0 kGy	2 kGy	4 kGy
1	0	6.61^d,x^	3.22^ab,y^	<2.0	6.60^e,x^	2.60^d,y^	<2.0	4.85^e,x^	<2.0	<2.0
	5	6.65^cd,x^	3.04^b,y^	<2.0	7.00^bc,x^	2.33^e,y^	<2.0	5.57^d,x^	<2.0	<2.0
	21	6.93^a,x^	2.28^f,y^	<2.0	6.97^cd,x^	2.54^d,y^	<2.0	5.95^cd,x^	<2.0	<2.0

7	0	6.70^bcd,x^	3.03^b,y^	<2.0	6.85^d,x^	2.97^b,y^	<2.0	6.13^c,x^	<2.0	<2.0
	5	6.87^ab,x^	2.74^cd,y^	<2.0	7.00^bc,x^	2.89^bc,y^	<2.0	5.98^c,x^	<2.0	<2.0
	21	6.64^d,x^	2.43^ef,y^	<2.0	7.11^ab,x^	2.07^f,y^	<2.0	6.32^bc,x^	<2.0	<2.0

14	0	6.86^abc,x^	3.25^a,y^	<2.0	7.07^bc,x^	3.22^a,y^	<2.0	6.82^a,x^	<2.0	<2.0
	5	6.63^d,x^	2.81^c,y^	<2.0	7.02^bc,x^	3.13^a,y^	<2.0	6.69^ab,x^	<2.0	<2.0
	21	6.86^abc,x^	2.58^de,y^	<2.0	7.21^a,x^	2.80^c,y^	<2.0	6.88^a,x^	<2.0	<2.0

Values with different superscript letters (a–f) within a column differ significantly (*P* < 0.05).

Values with different superscript letters (x–y) within a row for each pathogen differ significantly (*P* < 0.05).

<2.0: below detection limit.

*Target O_2_ levels in the original packages.

**Table 2:** Changes in* S. enteritidis*, *L. monocytogenes*, and *E. coli *O157:H7 counts (Log CFU g^−1^) in ready-to-cook meatballs during storage at 3°C as affected irradiation dose (kGy) and O_2_ level in 50% CO_2_ containing packages.

Storage (day)	O_2_*	*S. enteritidis*			*L. monocytogenes*			*E. coli* O157:H7		
	(%)	0 kGy	2 kGy	4 kGy	0 kGy	2 kGy	4 kGy	0 kGy	2 kGy	4 kGy
1	0	6.56^d,x^	3.07^b,y^	<2.0	6.92^b,x^	2.57^d,y^	<2.0	5.15^c,x^	<2.0	<2.0
	5	6.59^cd,x^	2.24^e,y^	<2.0	6.94^ab,x^	2.50^d,y^	<2.0	5.51^c,x^	<2.0	<2.0
	21	6.81^ab,x^	2.19^e,y^	<2.0	7.05^ab,x^	2.54^d,y^	<2.0	5.47^c,x^	<2.0	<2.0

7	0	6.83^ab,x^	3.05^b,y^	<2.0	6.92^b,x^	2.94^b,y^	<2.0	6.19^ab,x^	<2.0	<2.0
	5	6.83^ab,x^	2.98^bc,y^	<2.0	6.74^c,x^	2.70^cd,y^	<2.0	6.10^b,x^	<2.0	<2.0
	21	6.74^abc,x^	2.12^e,y^	<2.0	6.93^ab,x^	2.09^e,y^	<2.0	6.13^b,x^	<2.0	<2.0

14	0	6.89^a,x^	3.32^a,y^	<2.0	7.09^a,x^	3.20^a,y^	<2.0	6.58^a,x^	<2.0	<2.0
	5	6.64^bcd,x^	2.77^c,y^	<2.0	6.89^bc,x^	3.24^a,y^	<2.0	6.50^ab,x^	<2.0	<2.0
	21	6.86^a,x^	2.47^d,y^	<2.0	7.06^ab,x^	2.89^bc,y^	<2.0	6.50^ab,x^	<2.0	<2.0

Values with different superscript letters (a–d) within a column differ significantly (*P* < 0.05).

Values with different superscript letters (x–y) within a row for each pathogen differ significantly (*P* < 0.05).

<2.0: below detection limit.

*Target O_2_ levels in the original packages.

**Table 3:** D_10_-values (kGy) of the pathogens in ready-to-cook meatballs at 9°C as affected by the O_2_ levels.

O_2_ Level (%)	*E. coli* O157:H7	*S. enteritidis*	*L. monocytogenes*
0	0.40*	0.58	0.48
5	0.36*	0.50	0.44
21	0.35*	0.43	0.45

Average	0.37*	0.51	0.46

*Calculated by assuming complete inactivation of the pathogen by 2 kGy as no viable cells were detected in the irradiated samples.
